# Deciphering the Fine Details of C1 Assembly and Activation Mechanisms: “Mission Impossible”?

**DOI:** 10.3389/fimmu.2014.00565

**Published:** 2014-11-06

**Authors:** Christine Gaboriaud, Wai Li Ling, Nicole M. Thielens, Isabelle Bally, Véronique Rossi

**Affiliations:** ^1^Institut de Biologie Structurale, Université Grenoble Alpes, Grenoble, France; ^2^CNRS, Institut de Biologie Structurale, Grenoble, France; ^3^CEA, Institut de Biologie Structurale, Grenoble, France

**Keywords:** classical complement pathway, C1 complex, C1r, C1s, C1q, serine protease activation, complement-dependent cytotoxicity, X-ray structures

## Abstract

The classical complement pathway is initiated by the large (~800 kDa) and flexible multimeric C1 complex. Its catalytic function is triggered by the proteases hetero-tetramer C1r2s2, which is associated to the C1q sensing unit, a complex assembly of 18 chains built as a hexamer of heterotrimers. Initial pioneering studies gained insights into the main architectural principles of the C1 complex. A dissection strategy then provided the high-resolution structures of its main functional and/or structural building blocks, as well as structural details on some key protein–protein interactions. These past and current discoveries will be briefly summed up in order to address the question of what is still ill-defined. On a functional point of view, the main molecular determinants of C1 activation and its tight control will be delineated. The current perspective remains to decipher how C1 really works and is controlled *in vivo*, both in normal and pathological settings.

## Introduction

**Figure 1:**
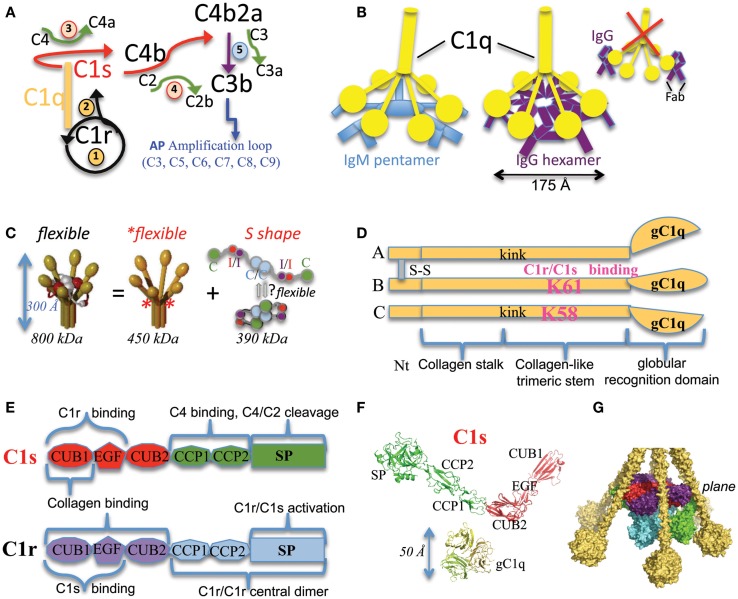
**Functional and structural elements of the CP activation**. **(A)** The main steps of the complement activation cascade through the CP. The multimeric C1q molecule is associated to the C1s–C1r–C1r–C1s tetramer. When C1q binds to an activating target surface, a conformational change triggers the auto-activation of the associated C1r protease (converting the pro-enzyme into an activated form, black circular arrow), which then activates C1s (black arrow). C1s activates C4 and C4b-bound C2 (red arrows), leading to the assembly of the classical C3 convertase C4b2a. Green arrows are used for the activation cleavage of C2, C3, and C4, with the release of a small fragment. Details of the consecutive AP amplification loop are not given for sake of clarity. It involves C3–C9 components and mediates rapid opsonization, signaling events, as well as eventually formation of the lytic pore. The initial steps are numbered from 1 to 5. The first two steps occur inside C1 and depend on C1q conformational change and the consequent C1r activation. Steps 3 and 4 depend on C1s proper positioning and catalytic activity. **(B)** Current hypothetical schemes on similar interaction modes between C1q and IgM or IgG hexamers, the best CP activators. The new scheme proposed for IgG is in contrast with the traditional old scheme (right) depicting one C1q molecule interacting with two distant IgG molecules, each antigen-bound through its two Fab arms. **(C)** The “C1 paradox” and initial low resolution C1 models. C1 is a 30 nm high multimer resulting from the association of the flexible recognition protein C1q with the flexible C1s–C1r–C1r–C1s tetramer, which appears more elongated (S extended shape) in solution than in the complex (thus the initial “paradox”). C1q (yellow) has a hexameric shape, built from 18 chains. Interaction (I) and catalytic (C) domains of C1r and C1s are labeled and colored on the right side. The asterisks show the position of flexible hinges in C1q. The low resolution model on the left and the proposed tetramer conformational equilibrium on the right are derived from (3). **(D)** Modular structure of each C1q chain type. A, B and C chains associate as a hexamer of ABC heterotrimers. Kink indicates the position of disruptions in the triplets occurring only within collagen-like sequences of the A and C chains and probably inducing flexible hinges. The disulfide bridging between chains A and B is illustrated. The C chain has no covalent link with A and B chains, but covalently associates pairs of ABC trimers through a C–C disulfide bridge. The two lysines crucial for C1 assembly are shown in pink. **(E)** Modular structure and associated functions of C1r and C1s. The catalytic domain includes the C-terminal serine protease (SP) domain as well as the preceding Complement Control Protein (CCP or sushi) modules. The interaction domains of C1s and C1r involve their N-terminal CUB–EGF–CUB modules. The corresponding functional implications are mentioned. The same color coding is used in **(F,G)** and in the right panel of **(C)**. “CUB” means initially found in Complement C1r and C1s, Uegf and BMP-1. **(F)** C1 is a large complex made of small building blocks of (mainly) known structures. The displayed C1s is a composite structure obtained after superposing the PDB structures 1ELV (4) and 4LMFA (5) onto 4LOT (5) (see details in Table S1 in Supplementary Material). The color code used is the same as in **(C,E)**. The chains ABC from the C1q globular domain [2WNV (6)] are shown on the same scale. **(G)** Example side view of a partial composite C1 model, refined using the results of differential accessibility in C1q and C1 using chemical lysine labeling followed by mass spectrometry (7). The C1r and C1s proteases interact with C1q through their interaction domains aligned on the same plane (which corresponds to the position of LysB61 and LysC58 in C1q). This part of the model is mainly confirmed by recent complementary experimental studies (8). The position of the catalytic domain of C1s is more uncertain and probably variable.

In 1897, at the very early period of nascent immunology, the Nobel price winner Jules Bordet discovered a heat-sensitive serum effector triggered by immune complexes and absolutely required for the lysis of Ab-coated erythrocytes or bacteria. At that time, it was named “alexine.” As discovered later on, this effector mechanism is very complex, involving many proteins, namely the complement system (C) triggered via the classical pathway (CP) (1, 2) (see Figures 1A,B). Deciphering the fine structural mechanisms governing this CP-activating function of the first C component C1 remains experimentally difficult and has progressed through iterative steps, which will be briefly summarized here.

Why is it important to decipher C1 structure and C activation mechanism? One obvious aim is to improve the C1-mediated effector mechanism in antibody therapeutics (8). C1 plays indeed a crucial role in the efficient elimination of Ab-coated targets, as confirmed by the disease susceptibility of patients affected by the deficiency in components C1q, C1r, C1s, and C4, all involved in the CP activation (9). Another hallmark of these deficiencies is the very large propensity to develop autoimmune diseases such as lupus erythematosus, which underlines that other essential functions are provided by the CP activation (9–13). On the other side, non-physiological activation of the CP or interferences by foreign substances such as carbon nanomaterial (14, 15) or a defective control of CP activation can also be strongly detrimental. Such undesirable activations can happen for example in cases of transplantation, neurological disorders, and rheumatoid arthritis (16) and thus new strategies to specifically inhibit the CP are awaited. On a more general standpoint, the functional impact of the complement system appears now far broader and more essential than initially assumed (17, 18).

## Initial Studies and First Low Resolution Functional C1 Models

Very active pioneering investigations were performed during the 1963–1987 period (1–3, 19). The sequences of the C1q, C1r, and C1s subcomponents, their fixed (C1q:C1r2s2) stoichiometry, as well as the calcium-dependency of the interaction between C1r and C1s have been deciphered. Biochemical experiments revealed that C1r and C1s are sequentially activated (Figure 1A) and their unique Arg-Ile activation cleavage site has been precisely identified (3). In both cases, a disulfide bridge maintains a covalent link between the catalytic serine protease (SP) domain and the preceding modules. Careful protein biochemical analyses detailed the numerous C1q post-translational modifications such as proline and lysine hydroxylations and hydroxylysine glycosylations, which were mainly confirmed recently (20). The main functional domains were isolated by limited proteolysis of the serum-derived proteins and their shape studied by several biophysical methods such as small angle X-ray or neutron scattering and electron microscopy (21–23) (see Figure 1C). C1q is a very flexible 450 kDa molecule, partly stabilized by the associated protease tetramer (24). Catalytic and interaction domains were identified for each C1r and C1s protease (Figure 1C). In an apparent paradox, a very elongated shape was observed by neutron scattering for the protease tetramer in solution (larger maximum radius of gyration Rg of 17 nm) in contrast to the measures for C1q (Rg of 12.8 nm) and for the C1 complex (Rg of 12.6 nm), which suggested a substantial conformational change of the tetramer and/or C1q upon association (Figure 1C) (3, 25). The other intriguing feature was about the symmetry level inside the complex since the C1q hexamer associates with a proteases tetramer (19, 24). Several “low resolution” models were proposed for C1 at that time, the main differences being the speculations about its activation mechanism and on how the proteases are tightly packed inside C1, and whether they are fully kept inside the C1q cone or not (3, 19, 24, 26).

## The Main Molecular Players Involved in C1 Activation and Its Tight Control

The C1r and C1s proteases are produced as inactive precursors (called zymogens), and thus need to be activated “on the spot” by a specific Arg-Ile proteolytic cleavage in response to a triggering signal. This activating cleavage induces a conformational rearrangement, as classically described for the proteases of the trypsin-like family. C1-inhibitor, a protease inhibitor of the serpin family, exerts the main physiological control on these C1r and C1s proteases activity, by both inhibiting their activation and dissociating them from activated C1. C1 auto-activation can be observed *in vitro* in the absence of C1 inhibitor or through heating, which induces large conformational changes and also probably kills the C1-inhibitory effect (19). The adverse effects related to uncontrolled C1 activation are thus mainly linked to unbalanced C1-inhibitor control. C1-inhibitor is a multipotent serpin, controlling also some proteases of the fibrinolytic system, and contact/kinin system of coagulation in addition to the C1r, C1s, and MASP complement proteases and thus its deficiency leads to severe diseases such as hereditary angioedema (27).

IgM or IgG immune complexes are the best physiological C1 activators identified to date, especially in the presence of C1-inhibitor. Although it has been known for long that C1q binds to IgG Fc domain, and that activation requires multivalent binding, the details of how this can happen had remained poorly understood (8). IgG mutations are known to strongly influence C1q binding and C activation (28–31). Of note, these mutation studies did not fully confirm the originally predicted E-x-K-x-K IgG C1q-binding consensus motif (28), which remains, however, still used by some teams as a C1q-binding predictive tool.

A recent study has shown how IgG surface clustering through Fc-dependent hexamers could lead to very efficient C1 activation (8) (Figure 1B). Interestingly, this mode of hexameric clustering is far more similar to the pentameric/hexameric IgM assembly than to what was traditionally proposed (Figure 1B). It has long been described in text books that C1 activation involves binding to at least two IgG molecules, each one bound to the surface through its two Fab segments (Figure 1B). In contrast, in the recently proposed hexameric IgG assembly, each IgG seems to have only one Fab arm on the target surface, the other Fab arm lying on the same central plane as the clustered Fc platform (8). This recent breakthrough brings new clues about how to enhance the complement-dependent cytotoxicity of IgG, since the E345R mutation was described as a general C1 activation enhancer for all IgG isotype variants (8). The recent structure of the deglycosylated IgG4 Fc further supports this hypothesis of a possible generic hexameric Fc assembly, which is stabilized by this E345R mutation (32). The IgG1 and IgG4 Fc form quite similar hexameric rings of 175 Å diameter, which is of the same range of magnitude as the 180 Å diameter estimated for the comparable IgM Cμ3–Cμ4 hexameric platform (32). Local differences are observed between the different IgG isotypes in their hexameric interface composition and surface loop conformations (32). Of note, the IgG4 homologous C1q-binding loop is flexible, with at least two different conformations observed. The major conformation observed in native IgG4 prevents C1q binding, which correlates with the strongly reduced level of CP activation by native IgG4 hexamers (32).

## Current Structural Knowledge on C1 Building Blocks and Key Protein–Protein Interactions

Although the first C1r crystals were obtained in 1981 [cited in Ref. (26)], X-ray crystallography analyses were initially limited, probably because of molecular flexibility. The C1 complex and most of its components look indeed very flexible (Figure 1C). A dissection strategy has thus been set up to determine the high-resolution structures of the main functional blocks (33) and of several structural joints, as detailed in Table S1 in Supplementary Material (Figures 1D–F). For the C1q molecule, only the X-ray structure of the C-terminal globular domain could be obtained (34), alone or in complex with minimal recognition motifs, such as deoxyribose for DNA, which gave insights into its recognition properties [reviewed previously in Ref. (35)].

More X-ray structures of C1r and C1s protease domains have been determined (Table S1 in Supplementary Material). The structures of all C1s modules are now known (Figure 1F). Detailed insights about conformational rearrangements were obtained by comparing different X-ray structures, for example between pro-enzyme and active states of the SP domains (36, 37), as well as some variations in inter-modular orientations (5, 38). The structure of the SP domains also revealed the main structural determinants of their restricted substrate specificity (4, 37, 38). However, C1s SP domain alone is not able to cleave C4 efficiently (39). C4 cleavage, which is the first step of both the classical and lectin activation pathways, appears thus to be more stringent since it requires additional exosites (40). The fine structural details about exosites in MASP-2 (the equivalent of C1s in the lectin pathway) and their interaction with C4 were unraveled recently (41). The functional implication of the homologous CCP exosite in C1s could be confirmed by mutational analyses (41). The structure of the C1s exosite at the CCP1/CCP2 interface was then solved recently (37). Interestingly, both the zymogen structure and surface plasmon resonance interaction analyses suggest that the C1s exosites are partly hidden in the pro-enzyme state (37).

**Figure 2:**
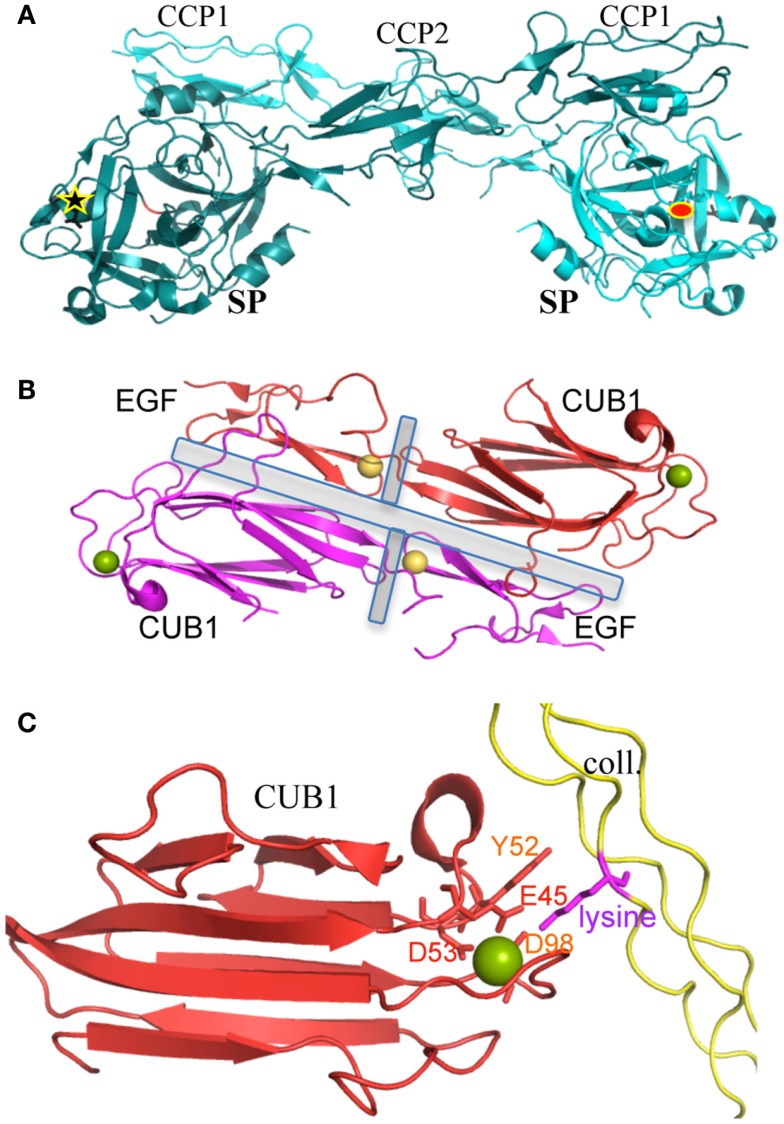
**Structures of key protein/protein interactions of C1 components**. **(A)** Dimeric association of the C1r catalytic domains. The interface involves similar interactions between the SP domain and the CCP1 module, in both the pro-enzyme and active C1r catalytic domain structures. This typical “butterfly” shape can be recognized on some electron micrographs from the proteases tetramer (23). The 90 Å distant active site (red ellipse) and activation site (black star) of the two molecules are highlighted [PDB code 1GPZ (36)]. **(B)** Dimeric CUB1–EGF interface (present *in vitro* in C1s homodimers and *in vivo* in C1r/C1s heterodimers). The central EGF calcium-binding sites stabilize both the inter- and intra-monomeric CUB-EGF interfaces (highlighted by gray rectangles). Since interface residues are mostly conserved in C1r (compared to C1s), we can assume that this head-to-tail packing observed with C1s homodimers also stands for the C1s–C1r heterodimer. This typical shape can also be recognized on some rare electron micrographs performed on the proteases tetramer (23). Yellow sphere, calcium in EGF; green sphere, calcium or magnesium in the C1s CUB1 module [PDB code 1NZI (45)]. **(C)** Calcium-dependent interaction between C1s CUB1 module and a lysine-containing collagen-like peptide [PDB code 4LOR (5)]. The main structural determinants are highlighted. The lysine side chain directly interacts with Glu45, Asp98, and Ser100. Asp53 is an essential component of the calcium-binding site. Mutations of Glu45, Tyr52, and Asp98 strongly alter C1q-binding properties [reviewed in Ref. (46)].

Structural details of protein–protein interactions relevant in terms of C1 assembly were also unraveled during this structural dissection, such as the head-to-tail interaction of the C1r catalytic domains. Such a dimeric interaction has been observed three times by X-ray crystallography and the butterfly-like side view (Figure 2A) can also be recognized at the center of early electron micrographs of the proteases tetramer (23, 36, 42). This interaction is maintained through contacts between the CCP1 module of one C1r subunit and the SP domain of its partner (36). One of the functional consequences is the larger than 90 Å distance between the active site of one monomer and the scissile bond of its partner, which prevents spontaneous mutual activation in this dimeric context (36). This auto-inhibited assembly looks like a “resting” state, which requires a conformational change to trigger C1 activation (36, 43). This interface between the catalytic domains of C1r is really specific of the CP activation, with no equivalent in the complexes activating the lectin pathway. Another structural feature of the C1r zymogen is the inactive occluded conformation of its primary binding site (44).

Calcium-dependent C1 assembly is controlled by the proteases CUB and EGF modules (47). The structural details governing these interactions have been mainly deciphered, although slightly indirectly. The C1r/C1s calcium-dependent interaction is mediated by their CUB1 and EGF modules, which form a head-to-tail dimer under the control of their EGF calcium-binding site (45) (Figure 2B). The calcium ion is tightly bound to the C1s EGF module in the context of the CUB1–EGF C1s dimeric interface, since it could not be replaced by lanthanides during soaking experiments used to solve the X-ray structure (45). This head-to-tail interaction can also be recognized on some early electron micrographs of the proteases tetramer (23). Unexpected calcium-binding sites are present in the CUB domains and govern the interactions between the proteases and the C1q collagen-like stems (45, 48). The calcium ion associated to the C1r CUB2 modules appears to be quite labile, although it greatly enhances the structural stability of these modules (49). Site-directed mutagenesis offered a very effective tool to confirm and detail the essential contributions of several amino acids in the full-length molecules: (i) It identified residues essential for C1q binding in C1r: E49, Y56, and D102 in CUB1; D226, H228, Y235, and D273 in CUB2. Other mutations severely affecting the C1q interactions were observed for E45 and Y52 in C1s CUB1 (46, 48). (ii) Conversely, the lysines B61 and C58 in C1q were identified by site-directed mutagenesis as essential protease-binding residues (50). These lysines are very close to the patient mutation GlyB63Ser resulting in a C1q functional deficiency including defective CP activation (12).

Similar CUB and EGF calcium-dependent interactions have then been observed in the MASPs-defense collagens complexes initiating the lectin complement pathway, as well as in other unrelated molecular systems (46, 51, 52). The structure of the C1s CUB1–EGF–CUB2 fragment in complex with a collagen-like fragment containing the OGKLGP sequence (O standing for hydroxyproline, Figure 2C) confirmed such a generic mode of association but reveals a different orientation of the CUB2 module as compared to MASP CUB1–EGF–CUB2 fragments (5).

## What Remains Still Ill-Defined?

Only the C1r CUB modules and the C1q collagen-like domain structures have not yet been solved at atomic resolution, but we know at least their overall shape and scaffold through homology and experimental analyses such as electron microscopy. The structure of the C1q recognition domain where the three subunits (Figure 1C) tightly interact with each other in a ACB clock-wise order (as seen from the collagen stem) has also indirectly given some clues about the relative ordering of the three chains in the preceding collagen-like stem (34, 47).

The isolated fragment X-ray structures or models can be combined into hypothetical C1 models (47). These C1-like models illustrate hypotheses in the 3D space about possible modes of C1 assembly and activation, which can then be further tested by site-directed mutagenesis (48). These models are idealized since, for example, C1 is always displayed as a symmetrical molecular complex although we know that it is highly flexible, which disrupts most of its symmetrical conformation in response to the environment. These models also aim to provide a synthetic overall representation consistent with accumulated experimental evidences (7). For example, the model depicted in Figure 1G accounted for the differential accessibility of lysine’s residues in C1q and C1 derived from mass spectrometry comparative analyses as well as previous experimental knowledge (7). However, such a dense C1 complex cannot be easily seen on electron microscopy images (unpublished results), and thus the corresponding C1 model remains an “*in silico*” interpretation (as well as most of C1 models).

Part of the “C1 paradox” has thus been elucidated since we know most of the building block structures and also key residues involved in C1 assembly, with now six C1q-binding sites in the protease tetramer (48). Nonetheless, details on how a flexible protease tetramer associates with such a flexible recognition molecule, and how C1 activation proceeds and is controlled remain ill-defined. In contrast to the *in vitro* studies, C1q and C1 can be found *in vivo* under flow conditions, both in the circulation and in the extra-vascular fluid, where shear stress could affect C1 assembly and activation (53). Moreover, observing fine structural details within C1 still represents a real experimental challenge because of its great flexibility and modular composition. The following questions are thus partially unanswered: How flexible is each inter-modular junction *in vivo*? Is the C1r CUB2 module only partially saturated by calcium *in vivo*, and thus possibly marginally stable within C1 (49)? What is the role of the charged and flexible long insertion in C1r EGF (54)? Which chain is at the leading, medium, and edge position in the native C1q collagen heterotrimeric stem? What are the relative positions between these native C1q stems and the proteases CUB domains? Do the proteases stably stay attached to C1q or is there a fast assembly/disassembly equilibrium? What drives the spectacular conformational change of the proteases from their elongated flexible shape in solution toward the assumed compact C1-associated conformation? How can we observe, describe, or deduce the details of the conformational changes involved during C1 activation? How can we observe the transmission of the triggering signal from C1q recognition to C1r activation? How can we characterize the required C1q conformational change(s)? How is C1r activation propagated to the successive C1s, C4, and C2 activations? How does C1-inhibitor finely control these processes? What about C1 activation by non-immune targets in a physiological or pathological context? How do differences in antigenic structures and surface density precisely modulate the levels of CP activation by the Ab-coated targets? How can we predict the classical C activation outcome when C1q binds to ligands through its globular heads? How do pathogens interfere with C1 activation?

## Perspectives

Over the years, detail after detail, the image describing the immunoglobulins/C1 interaction is gradually emerging. But the flexibility of the C1 molecule and its thin flexible building elements such as the collagen-like stalks make its fine details difficult to observe. Even electron microscopy performed on C1 bound to hexameric IgG surface clusters on liposomes did not fully overcome the limitations due to C1 flexibility, since only four (out of the six expected) globular densities probably corresponding to C1q recognition domains could be consistently observed on top of the hexameric IgG assembly (8). The collagen stems are also too thin, fragile, and flexible to be seen on averaged density maps. Only the position of the larger N-terminal collagen stalk remains visible after averaging. Visible density also remains after averaging for the region probably corresponding to the interaction domains of C1r and C1s, which fill a continuous section inside the C1q cone.

In conclusion, although refining the structural details of C1 assembly and activation remains a difficult challenge, this mission does not sound definitively “impossible.” The scientific community will probably find out new solutions to further decrypt the fine structural details, for example by matching X-ray structures and electron density maps obtained from new developments in electron microscopy and associated computing strategies. The use of recombinant C1 fragments (C1q, C1r, C1s) will be useful to further check in detail their structure/function relationships.

## Conflict of Interest Statement

The authors declare that the research was conducted in the absence of any commercial or financial relationships that could be construed as a potential conflict of interest.

## Supplementary Material

The Supplementary Material for this article can be found online at http://www.frontiersin.org/Journal/10.3389/fimmu.2014.00565/abstract
