# Supplementary material for: Deciphering the Fine Details of C1 Assembly and Activation Mechanisms: “Mission Impossible”?
Source: Front Immunol. 2014 Nov 6;5:565. doi: 10.3389/fimmu.2014.00565 (PMC4222235; doi:10.3389/fimmu.2014.00565)
Supplement: Supplementary file 1 [file Table_1.DOCX]

***Supplementary Material***

**Deciphering the fine details of C1 assembly and activation mechanisms: 'mission impossible'?**

**Christine Gaboriaud ^123^, Wai Li Ling ^123^, Nicole M. Thielens ^123^, Isabelle Bally ^123^, Véronique Rossi ^123^**

^1^ IBS, Univ. Grenoble Alpes, F-38044 Grenoble, France

^2^ IBS, CNRS, F-38044 Grenoble, France

^3^ IBS, CEA, F-38044 Grenoble, France

*** Correspondence:** Gaboriaud Christine, IBS, Grenoble, France

christine.gaboriaud@ibs.fr

**Supplementary Table 1. X-ray/NMR structures of C1-related modules/fragments/complexes**

| Module/Domain | type | PDB code | Resol. (Å) | Year | Ref. |
| --- | --- | --- | --- | --- | --- |
| C1q globular domain | F | 1PK6 | 1.85 | 2003 | (1) |
| C1q globular domain/dR | C | 2WNV | 1.25 | 2010 | (2) |
| C1q globular domain/hS | C | 2WNU | 2.30 | 2010 | (2) |
| C1q globular domain/PS | C | 2JG8 | 2.05 | 2008 | (3) |
| C1q globular domain | F | 2JG9 | 1.90 | 2008 | (3) |
| C1s CUB1-EGF-CUB2+ C1q-like collagen peptide | A/C/M | 4LOR | 2.50 | 2013 | (4) |
| C1s CUB2-CCP1-CCP2 | L | 4LOT | 2.92 | 2013 | (4) |
| C1s CUB2-CCP1 | L | 4LOS | 2.00 | 2013 | (4) |
| C1s CUB1-EGF-CUB2 | A | 4LMF | 2.92 | 2013 | (4) |
| C1s CCP2-SP | F | 1ELV | 1.70 | 2000 | (5) |
| C1s CUB1-EGF | A | 1NZI | 1.50 | 2003 | (6) |
| C1s CCP1-CCP2-SP | Z | 4J1Y | 2.66 | 2013 | (7) |
| C1r EGF | I | 1APQ | NMR | 1997 | (8) |
| C1r CCP1-CCP2-SP | Z/M/C | 1GPZ | 2.90 | 2002 | (9) |
| C1r CCP2-SP | Z/M | 1MD7 | 3.20 | 2002 | (10) |
| C1r CCP2-SP | F | 1MDA | 2.80 | 2002 | (10) |
| C1r CCP1-CCP2-SP | F/C | 2QY0 | 2.60 | 2008 | (11) |

Type abbreviation: **F**unctional (recognition domain or active catalytic domain), **C**omplex, **Z**ymogen (inactive precursor), **A**ssociation domain, **I**solated module, **M**odified mutant form;

Ligand abbreviation: hS: heparan sulfate; dR: deoxyribose: PS: phosphoserine.

**References**

1. Gaboriaud C, Juanhuix J, Gruez A, Lacroix M, Darnault C, Pignol D, Verger D, Fontecilla-Camps JC, Arlaud GJ. The crystal structure of the globular head of complement protein C1q provides a basis for its versatile recognition properties. *J Biol Chem* (2003) 278:46974–46982. doi:10.1074/jbc.M307764200

2. Garlatti V, Chouquet A, Lunardi T, Vivès R, Païdassi H, Lortat-Jacob H, Thielens NM, Arlaud GJ, Gaboriaud C. Cutting edge: C1q binds deoxyribose and heparan sulfate through neighboring sites of its recognition domain. *J Immunol Baltim Md 1950* (2010) 185:808–812. doi:10.4049/jimmunol.1000184

3. Païdassi H, Tacnet-Delorme P, Garlatti V, Darnault C, Ghebrehiwet B, Gaboriaud C, Arlaud GJ, Frachet P. C1q binds phosphatidylserine and likely acts as a multiligand-bridging molecule in apoptotic cell recognition. *J Immunol Baltim Md 1950* (2008) 180:2329–2338.

4. Venkatraman Girija U, Gingras AR, Marshall JE, Panchal R, Sheikh MA, Gal P, Schwaeble WJ, Mitchell DA, Moody PCE, Wallis R. Structural basis of the C1q/C1s interaction and its central role in assembly of the C1 complex of complement activation. *Proc Natl Acad Sci* (2013) 110:13916–13920. doi:10.1073/pnas.1311113110

5. Gaboriaud C, Rossi V, Bally I, Arlaud GJ, Fontecilla-Camps JC. Crystal structure of the catalytic domain of human complement c1s: a serine protease with a handle. *EMBO J* (2000) 19:1755–1765. doi:10.1093/emboj/19.8.1755

6. Gregory LA, Thielens NM, Arlaud GJ, Fontecilla-Camps JC, Gaboriaud C. X-ray structure of the Ca2+-binding interaction domain of C1s. Insights into the assembly of the C1 complex of complement. *J Biol Chem* (2003) 278:32157–32164. doi:10.1074/jbc.M305175200

7. Perry AJ, Wijeyewickrema LC, Wilmann PG, Gunzburg MJ, D’Andrea L, Irving JA, Pang SS, Duncan RC, Wilce JA, Whisstock JC, et al. A molecular switch governs the interaction between the human complement protease C1s and its substrate, complement C4. *J Biol Chem* (2013) 288:15821–15829. doi:10.1074/jbc.M113.464545

8. Bersch B, Hernandez JF, Marion D, Arlaud GJ. Solution structure of the epidermal growth factor (EGF)-like module of human complement protease C1r, an atypical member of the EGF family. *Biochemistry (Mosc)* (1998) 37:1204–1214. doi:10.1021/bi971851v

9. Budayova-Spano M, Lacroix M, Thielens NM, Arlaud GJ, Fontecilla-Camps JC, Gaboriaud C. The crystal structure of the zymogen catalytic domain of complement protease C1r reveals that a disruptive mechanical stress is required to trigger activation of the C1 complex. *EMBO J* (2002) 21:231–239. doi:10.1093/emboj/21.3.231

10. Budayova-Spano M, Grabarse W, Thielens NM, Hillen H, Lacroix M, Schmidt M, Fontecilla-Camps JC, Arlaud GJ, Gaboriaud C. Monomeric structures of the zymogen and active catalytic domain of complement protease c1r: further insights into the c1 activation mechanism. *Struct Lond Engl 1993* (2002) 10:1509–1519.

11. Kardos J, Harmat V, Palló A, Barabás O, Szilágyi K, Gráf L, Náray-Szabó G, Goto Y, Závodszky P, Gál P. Revisiting the mechanism of the autoactivation of the complement protease C1r in the C1 complex: structure of the active catalytic region of C1r. *Mol Immunol* (2008) 45:1752–1760. doi:10.1016/j.molimm.2007.09.031
